# Supplementary material for: Hypertension-Related Status and Influencing Factors among Chinese Children and Adolescents Aged 6~17 Years: Data from China Nutrition and Health Surveillance (2015–2017)
Source: Nutrients. 2024 Aug 13;16(16):2685. doi: 10.3390/nu16162685 (PMC11357336; doi:10.3390/nu16162685)
Supplement: Supplementary file 1 [file nutrients-16-02685-s001.zip › nutrients-3139713-supplementary.pdf]

Supplementary Table S1. Weighted blood pressure level among Chinese children and adolescents 6~17 years of age by age–group.

Age group	SBP (mmHg)			*P* value	DBP (mmHg)			*P* value
	Total	Males	Females		Total	Males	Females	
06–	104.4 (103.3–105.6)	105.2 (104.0–106.4)	103.6 (102.4–104.8)	0.0003	64.0 (62.7–65.4)	63.9 (62.6–65.3)	64.1 (62.6–65.6)	0.6865
07–	105.1 (104.2–106.1)	105.7 (104.7–106.6)	104.5 (103.5–105.6)	0.0016	63.5 (62.6–64.5)	63.2 (62.2–64.2)	63.9 (62.9–64.8)	0.0355
08–	107.2 (106.2–108.1)	108.3 (107.4–109.2)	105.9 (104.7–107.0)	< 0.0001	64.9 (64.0–65.9)	65.0 (64.0–66.0)	64.9 (63.8–65.9)	0.8562
09–	107.9 (106.9–108.9)	108.6 (107.5–109.6)	107.0 (106.0–108.0)	< 0.0001	65.4 (64.5–66.2)	65.7 (64.6–66.7)	65.0 (64.1–65.8)	0.0856
10–	109.7 (108.7–110.6)	109.7 (108.8–110.7)	109.6 (108.4–110.8)	0.8167	66.1 (65.3–66.9)	65.8 (64.8–66.8)	66.4 (65.6–67.2)	0.1626
11–	111.9 (110.9–112.8)	112.1 (111.0–113.3)	111.6 (110.6–112.5)	0.1799	67.1 (66.3–67.8)	66.9 (66.1–67.8)	67.2 (66.4–68.0)	0.4044
12–	112.6 (111.7–113.6)	113.3 (112.4–114.3)	111.9 (110.8–112.9)	0.0005	66.8 (66.1–67.5)	66.5 (65.9–67.2)	67.1 (66.3–68.0)	0.0498
13–	114.7 (113.9–115.5)	116.1 (114.9–117.3)	113.1 (112.2–114.0)	< 0.0001	67.1 (66.5–67.7)	66.4 (65.8–67.1)	67.9 (67.3–68.6)	< 0.0001
14–	116.0 (115.0–117.0)	118.3 (117.0–119.6)	113.1 (112.1–114.0)	< 0.0001	67.7 (67.0–68.4)	67.3 (66.4–68.2)	68.2 (67.4–68.9)	0.0448
15–	115.7 (114.8–116.6)	119.5 (118.4–120.5)	112.1 (111.1–113.1)	< 0.0001	67.6 (66.9–68.2)	67.5 (66.7–68.3)	67.6 (66.9–68.4)	0.6705
16–	116.7 (116.0–117.5)	120.9 (120.0–121.8)	112.4 (111.5–113.2)	< 0.0001	68.3 (67.7–68.8)	68.3 (67.7–68.9)	68.2 (67.6–68.8)	0.7501
17–18	117.1 (116.1–118.0)	120.6 (119.4–121.8)	112.4 (111.3–113.4)	< 0.0001	68.6 (68.0–69.2)	68.8 (68.1–69.6)	68.3 (67.3–69.2)	0.2858

Data are represented as means (95% CI).

Supplementary Table S2. Crude prevalence and weighted prevalence of HTN and pre–HTN by age group.

Age group, y	N	HTN			*P* value	Pre–HTN			*P* value
		Total	Males	Females		Total	Males	Females	
Crude prevalence									
Overall	67,947	24.3 (24.0–24.6)	23.9 (23.4–24.3)	24.7 (24.2–25.2)	0.0112	15.8 (15.6–16.1)	17.9 (17.5–18.3)	13.8 (13.4–14.2)	<0.0001
06–	3,462	30.4 (28.9–31.9)	29.9 (27.7–32.1)	30.8 (28.7–33.0)	0.5462	13.1 (11.9–14.2)	13.5 (11.9–15.3)	12.6 (11.1–14.2)	0.4931
07–	6,596	27.4 (26.4–28.5)	26.6 (25.1–28.1)	28.2 (26.7–29.8)	0.1456	12.4 (11.6–13.2)	12.3 (11.2–13.4)	12.5 (11.4–13.7)	0.4833
08–	6,953	27.7 (26.7–28.8)	28.1 (26.6–29.6)	27.4 (25.9–28.8)	0.4801	13.8 (13.0–14.6)	13.6 (12.4–14.7)	14.0 (12.8–15.1)	0.7498
09–	6,995	25.7 (24.7–26.7)	25.4 (23.9–26.8)	26.1 (24.6–27.5)	0.4980	14.1 (13.3–14.9)	13.5 (12.4–14.6)	14.7 (13.5–15.9）	0.0964
10–	6,906	23.9 (22.8–24.9)	22.9 (21.5–24.3)	24.8 (23.4–26.3)	0.0590	14.0 (13.1–14.8)	13.9 (12.8–15.2)	14.0 (12.8–15.2)	0.6266
11–	6,917	24.8 (23.7–25.8)	23.3 (21.9–24.7)	26.3 (24.8–27.8)	0.0040	14.2 (13.4–15.0)	14.5 (13.3–15.7)	13.9 (12.8–15.1)	0.9916
12–	7,078	22.4 (21.4–23.4)	20.9 (19.5–22.3)	23.9 (22.5–25.3)	0.0022	15.2 (14.4–16.0)	15.8 (14.6–17.1)	14.5 (13.4–15.7)	0.411
13–	6,343	22.3 (21.3–23.3)	20.6 (19.2–22.1)	24.0 (22.5–25.5)	0.0014	17.0 (16.1–17.9)	19.7 (18.3–21.1)	14.2 (13.0–15.5)	<0.0001
14–	3,748	22.3 (21.0–23.7)	20.6 (18.8–22.5)	24.1 (22.2–26.0)	0.0105	18.2 (17.0–19.5)	23.4 (21.5–25.4)	12.9 (11.4–14.5)	<0.0001
15–	4,462	20.8 (19.6–22.0)	22.1 (20.3–23.9)	19.6 (18.0–21.2)	0.0384	20.3 (19.2–21.5)	27.6 (25.7–29.5)	13.7 (12.3–15.1)	<0.0001
16–	6,253	21.4 (20.4–22.4)	23.9 (22.4–25.4)	18.9 (17.6–20.4)	<0.0001	21.6 (20.6–22.7)	29.5 (27.9–31.1)	13.9 (12.7–15.1)	<0.0001
17–18	2,234	20.1 (18.5–21.9)	21.4 (19.1–23.8)	18.8 (16.4–21.3)	0.1254	23.1 (21.4–24.9)	31.4 (28.8–34.1)	13.6 (11.5–15.8)	<0.0001
Weighted prevalence									
Overall	67,947	24.9 (22.7–27.2)	25.0 (22.8–27.3)	24.8 (22.3–27.2)	0.6866	17.1 (16.1–18.0)	19.7 (18.3–21.0)	14.1 (13.3–15.0)	<0.0001
06–	3,462	35.2 (29.3–41.1)	34.8 (29.2–40.3)	35.6 (28.0–43.3)	0.5462	13.0 (10.6–15.5)	13.8 (10.0–17.7)	12.1 (9.5–14.7)	0.3979
07–	6,596	30.5 (26.5–34.5)	29.0 (25.1–32.9)	32.3 (27.4–37.3)	0.1456	13.3 (11.7–14.8)	13.6 (11.7–15.6)	12.8 (10.6–15.1)	0.9164
08–	6,953	30.7 (27.0–34.4)	30.9 (26.9–34.8)	30.6 (26.2–35.0)	0.4801	13.5 (11.8–15.2)	14.1 (11.9–16.4)	12.7 (10.7–14.8)	0.2717
09–	6,995	27.3 (23.3–31.3)	27.0 (22.5–31.5)	27.8 (23.8–31.8)	0.4980	14.3 (12.6–15.9)	13.8 (11.6–16.0)	14.8 (12.8–16.9)	0.3958
10–	6,906	26.1 (22.5–29.6)	25.0 (21.3–28.7)	27.3 (22.4–32.2)	0.0590	14.8 (12.9–16.6)	13.9 (11.0–16.8)	15.8 (13.3–18.3)	0.2941
11–	6,917	26.9 (23.5–30.4)	26.9 (23.4–30.4)	27.0 (23.1–30.9)	0.0040	13.6 (12.0–15.2)	13.6 (11.8–15.5)	13.6 (11.2–16.0)	0.9633
12–	7,078	22.6 (19.8–25.4)	22.2 (19.3–25.1)	23.1 (19.4–26.8)	0.0022	16.4 (14.7–18.1)	16.9 (15.0–18.8)	15.8 (13.6–18.0)	0.4024
13–	6,343	21.6 (19.0–24.2)	20.2 (17.3–23.2)	23.2 (20.2–26.2)	0.0014	18.7 (16.9–20.5)	19.9 (17.1–22.7)	17.3 (13.6–21.1)	0.5051

14–	3,748	23.3 (19.5–27.1)	21.9 (17.4–26.4)	25.1 (21.4–28.7)	0.0105	19.3 (16.7–21.9)	24.7 (21.4–28.1)	12.5 (9.7–15.2)	<0.0001
15–	4,462	20.3 (17.4–23.2)	22.5 (18.9–26.0)	18.2 (14.7–21.6)	0.0384	20.2 (18.3–22.1)	26.8 (23.4–30.1)	13.8 (11.4–16.3)	<0.0001
16–	6,253	20.8 (18.3–23.2)	23.0 (20.2–25.8)	18.5 (15.3–21.6)	<0.0001	21.6 (19.6–23.6)	29.9 (27.0–32.8)	12.9 (11.0–14.7)	<0.0001
17–18	2,234	18.5 (15.9–21.0)	19.3 (16.2–22.4)	17.4 (13.9–20.9)	0.1254	26.2 (22.8–29.5)	34.4 (29.8–38.9)	15.3 (11.9–18.6)	<0.0001

Data are represented as percentage (95% CI).

Supplementary Table S3. Multivariable-adjusted odds ratios for HTN and pre-HTN.

Characteristics	HTN			Pre-HTN		
	Total	Males	Females	Total	Males	Females
Gender (Females vs. Males)	1.09 (1.01–1.18) *	-	-	0.66 (0.60–0.73) *	-	-
Age group (12 ~ 17 vs. 6 ~ 11 years)	0.79 (0.69–0.91) *	0.82 (0.70–0.95) *	0.76 (0.64–0.91) *	1.42 (1.25–1.61) *	1.83 (1.53–2.19) *	1.02 (0.86–1.22) *
BMI						
Normal	Reference	Reference	Reference	Reference	Reference	Reference
Overweight	1.73 (1.49–1.99) *	1.69 (1.39–2.06) *	1.74 (1.51–2.00) *	1.78 (1.57–2.03) *	1.80 (1.53–2.12) *	1.64 (1.33–2.02) *
Obese	3.30 (2.80–3.88) *	3.34 (2.71–4.11) *	3.07 (2.52–3.73) *	2.07 (1.77–2.42) *	2.10 (1.69–2.61) *	1.86 (1.39–2.48) *
Central obesity (Yes vs. No)	1.23 (1.08–1.41) *	1.32 (1.11–1.57) *	1.15 (0.99–1.35)	1.03 (0.92–1.16)	1.14 (0.98–1.32)	0.98 (0.82–1.18)
Living area (Rural vs. Urban)	1.28 (1.04–1.57) *	1.22 (1.00–1.50)	1.34 (1.05–1.70) *	1.17 (0.99–1.39)	1.06 (0.86–1.31)	1.34 (1.13–1.58) *
Geographical region						
East	Reference	Reference	Reference	Reference	Reference	Reference
Central	0.99 (0.78–1.27)	1.00 (0.79–1.27)	0.98 (0.74–1.30)	1.16 (0.97–1.40)	1.16 (0.93–1.46)	1.13 (0.93–1.38)
West	0.76 (0.57–1.01)	0.76 (0.56–1.03)	0.76 (0.56–1.02)	0.89 (0.73–1.10)	0.88 (0.69–1.11)	0.90 (0.73–1.11)
Maternal education level						
Primary school or below	Reference	Reference	Reference	Reference	Reference	Reference
Junior middle school	0.95 (0.84–1.08)	0.92 (0.77–1.09)	0.99 (0.87–1.11)	1.02 (0.93–1.11)	0.99 (0.87–1.13)	1.05 (0.91–1.21)
High school or higher	0.93 (0.79–1.09)	0.88 (0.73–1.07)	0.97 (0.81–1.17)	1.02 (0.88–1.17)	0.99 (0.82–1.19)	1.05 (0.86–1.28)
Household income per capita						
< 10,000	Reference	Reference	Reference	Reference	Reference	Reference
10,000 ~	0.90 (0.78–1.04)	0.85 (0.71–1.02)	0.95 (0.79–1.13)	0.85 (0.71–1.01)	0.86 (0.68–1.09)	0.80 (0.66–0.97) *
> 25,000	0.79 (0.67–0.94) *	0.79 (0.62–0.99)	0.79 (0.60–1.03)	1.08 (0.84–1.38)	1.14 (0.84–1.55)	0.93 (0.70–1.24)
Not given	0.82 (0.68–0.99) *	0.82 (0.67–1.00)	0.81 (0.65–1.01)	0.90 (0.78–1.05)	0.90 (0.74–1.11)	0.87 (0.74–1.03)
Physical activity (Inadequate vs. Adequate)	1.12 (0.97–1.30)	1.09 (0.92–1.29)	1.17 (1.01–1.37) *	1.02 (0.92–1.13)	1.10 (0.97–1.24)	1.00 (0.88–1.13)
Video time (> 2h vs. ≤ 2h)	0.94 (0.85–1.03)	0.95 (0.84–1.07)	0.93 (0.83–1.04)	1.04 (0.93–1.16)	0.99 (0.85–1.16)	1.11 (0.99–1.25)
Sleep duration (h)						
7 ~	Reference	Reference	Reference	Reference	Reference	Reference

< 7	0.99 (0.82–1.19)	1.17 (0.92–1.50)	0.85 (0.64–1.13)	1.17 (0.95–1.46)	1.37 (1.03–1.82) *	1.07 (0.84–1.36)
≥ 9	1.26 (1.13–1.40) *	1.20 (1.05–1.38) *	1.32 (1.16–1.51) *	0.94 (0.85–1.04)	0.82 (0.72–0.93) *	1.14 (0.98–1.32)
Family history of HTN (Yes vs. No)	0.96 (0.88–1.04)	0.95 (0.85–1.06)	0.98 (0.87–1.10)	1.03 (0.95–1.12)	1.13 (1.03–1.25) *	0.93 (0.82–1.07)
Second-hand smoking exposure (Yes vs. No)	1.00 (0.91–1.10)	1.11 (0.97–1.26)	0.89 (0.79–1.00)	0.99 (0.91–1.07)	0.98 (0.88–1.10)	0.96 (0.84–1.09)
DASH score						
Q1	Reference	Reference	Reference	Reference	Reference	Reference
Q2	0.97 (0.85–1.10)	0.99 (0.83–1.18)	0.95 (0.83–1.10)	1.11 (0.98–1.27)	1.05 (0.90–1.21)	1.25 (1.02–1.53) *
Q3	0.92 (0.80–1.07)	0.94 (0.79–1.12)	0.92 (0.78–1.08)	1.05 (0.95–1.16)	0.99 (0.88–1.12)	1.20 (1.03–1.40) *
Q4	0.99 (0.85–1.14)	1.01 (0.84–1.21)	0.97 (0.83–1.15)	1.09 (0.95–1.24)	1.10 (0.93–1.31)	1.12 (0.94–1.34)

Data are represented as OR (95% CI).

* Indicated *p* value < 0.05.
